# Psychopathy and Corticostriatal Connectivity: The Link to Criminal Behavior in Methamphetamine Dependence

**DOI:** 10.3389/fpsyt.2020.00090

**Published:** 2020-02-28

**Authors:** William F. Hoffman, Merel B. Jacobs, Laura E. Dennis, Holly D. McCready, Alex W. Hickok, Sheehan B. Smith, Milky Kohno

**Affiliations:** ^1^ Mental Health Division P35C, Veterans Affairs Portland Health Care System, Portland, OR, United States; ^2^ Department of Psychiatry, Oregon Health & Science University, Portland, OR, United States; ^3^ Department of Behavioral Neuroscience, Oregon Health & Science University, Portland, OR, United States; ^4^ Methamphetamine Abuse Research Center (MARC), Oregon Health & Science University and Veterans Affairs Portland Health Care System, Portland, OR, United States; ^5^ Center of Innovation to Improve Veteran Involvement in their Care, VA Portland VA Healthcare System, Portland, OR, United States

**Keywords:** methamphetamine, resting state – fMRI, psychopathy (PPI-R), corticostriatal, ventral striatum

## Abstract

Methamphetamine use and psychopathy are associated with criminal behavior; however, it is unclear how methamphetamine use and psychopathy interact to promote violent, economic and drug offenses. Abnormalities in corticostriatal functional connectivity are exhibited in both psychopathic and methamphetamine dependent individuals, which may contribute to criminal behavior through maladaptive and impulsive decision-making processes. This study shows that psychopathic traits contribute to weaker corticostriatal connectivity in methamphetamine dependence and contributes to an increase in criminal behavior. As the propensity to engage in criminal activity is dependent on a number of factors, a hierarchical regression identifies the contribution of the impulsive antisocial domain of psychopathy, anxiety, years of methamphetamine use and corticostriatal connectivity on different types of criminal offenses. Methamphetamine use and psychopathic traits reduce treatment responsiveness and increase the likelihood of recidivism, and it is therefore important to understand the factors underlying the propensity to engage in criminal behavior.

## Introduction

Substance use is especially prevalent among individuals with co-occurring psychiatric conditions, including psychopathic personality traits. Although psychopathy only affects approximately 1% of the population, antisocial diagnoses are greater in individuals with substance-use disorders, and are associated with early onset of drug use (1] and with development of polysubstance dependence (2, 3). Psychopathy and addiction share common behavioral phenotypes, including poor behavioral control, impulsivity, novelty seeking and risk-taking (4–11), which may contribute to an increase in criminal activities in both populations (10, 12). Although all drugs of abuse are associated to some extent with criminal activities, methamphetamine (MA) users are more likely than other drug users to commit acts of violence (10). MA users also exhibit heightened categorical and dimensional aspects of antisocial and psychopathic traits, including interpersonal violence, hostility, and aggression (13, 14). There have been no studies, however, investigating the complex interaction of psychopathy in MA use disorder and its effect on criminal behavior.

Results from neuroimaging studies on psychopathy and MA use disorder converge on a set of brain regions responsible for emotional processing and behavioral planning, including the insula, amygdala, dorsal/ventral striatum, anterior cingulate cortex, and the dorsolateral and orbital/ventral medial prefrontal cortex (PFC) (15, 16). Notably, abnormalities in ventral striatal function and corticostriatal resting-state functional connectivity (RSFC) are similar in psychopathy and addiction. Individuals with psychopathic traits and individuals with MA use disorder have increased ventral striatal response to reward during temporal discounting (9) and risky decision-making (5), respectively. Resting-state functional connectivity results also show similar patterns, where psychopathy and MA dependence are both associated with abnormalities in corticostriatal RSFC (5, 9, 17, 18). As disruptions in striatal function and corticostriatal connectivity are linked with poor behavioral regulation and impulsiveness (4), the integrity of these regions may be critical in controlling various signals that increase the propensity for aggression and violence and shape maladaptive criminal behaviors.

Psychopathic traits and the link to criminal behavior is important to understand, from both substance abuse treatment and criminal justice perspectives, as individuals with histories of antisocial behavior are more likely to re-offend and less likely to successfully complete substance abuse treatment (10). A number of studies have examined how different dimensions of personality may predispose individuals to engage in criminal activities. Results suggest that psychopathy and anxiety are common traits in criminal offenders, where high levels of anxiety, nervous tension, and distress on the Karolinska Scales of Personality (KSP) were reported in a group of 130 individuals convicted of serious criminal offenses (19). Similarly, a cohort of criminally convicted women reported significantly high levels of anxiety, irritability, hostility, and insecurity, and individuals convicted of murder self-report significantly high levels on anxiety measures and on obsessive and psychopathic traits (20). These studies suggest a strong link between criminal behavior and anxious and psychopathic traits, and although some studies find a negative association between psychopathy and anxiety, anxiety is positively related to the anti-social impulsive dimensions of psychopathy (8, 21, 22).

Taken together, these studies provide evidence for a link between criminal behavior, brain function, and personality traits, however there has been no systematic study on the mechanism by which these processes promote criminal behavior in MA use disorder. Given that inhibitory control deficits are linked to corticostriatal function, which is compromised in both psychopathy and addiction, this study aims to examine the associations between brain connectivity, psychopathy, generalized anxiety, and criminal behavior in individuals with MA dependence. Both psychopathy and MA use has been linked to a decrease in corticostriatal connectivity but an increase in mesolimbic dopamine function and activation of the ventral striatal in response to rewards (5, 9). We therefore expect that the MA group will exhibit a negative relationship between psychopathy scores and connectivity between the ventral striatum and prefrontal cortex. As anxiety and psychopathy are linked to criminal behavior, we hypothesize that the increase in criminal behavior in the MA group will be associated with higher levels of anxiety and psychopathic traits. A systematic evaluation of the association of criminal behavior with brain function and personality traits in MA dependence is critical in addressing the clinical course and response to appropriately matched treatments, which may reduce incarceration.

## Methods

### Participants

Thirty-three volunteers diagnosed with MA dependence (DSM-IV), recruited from the VA Portland Healthcare System (VAPORHCS) and community substance abuse treatment programs and 38 healthy controls recruited with online advertisements, provided written informed consent, as approved by the VAPORHCS and Oregon Health & Science University Institutional Review Boards. Exclusion criteria, determined by medical history and laboratory blood tests were: systemic, neurological, cardiovascular, or pulmonary disease, head trauma with loss of consciousness, magnetic resonance imaging (MRI) contraindications, use of medications known to have dopaminergic mechanisms (e.g., antipsychotics, antidepressants, antiparkinsonian agents), sedative-hypnotics (barbiturates, benzodiazepines, zolpidem), or anticholinergics. Past or Current Axis I diagnoses, other than MA dependence (MA group) or nicotine dependence (either group), assessed with the Structured Clinical Inventory for DSM-IV-TR, were exclusionary. Urine testing on day of MRI scan verified abstinence from cocaine, methamphetamine, benzodiazepines, opiates, and cannabinoids. Participants were able to smoke cigarettes up to 1 h before scanning to minimize and balance the effects of recent smoking on brain function against the effects of nicotine withdrawal and craving.

### Neuropsychiatric and Criminal History Interview

The Psychopathic Personality Inventory - Revised (PPI-R) is a 154-item self-report scale that assesses psychopathy by measuring 8 factors that index psychopathic personality traits including, Machiavellian Egocentricity, Rebellious Nonconformity, Blame Externalization, Carefree Nonplanfulness, Social Influence, Fearlessness, Stress Immunity, and Coldheartedness. The PPI-R is composed of two underlying latent factors; PPI-1: Fearless Dominance and PPI-2: Impulsive Antisocial. The PPI-1 is a composite score of Social Influence, Fearlessness, and Stress Immunity, and higher scores are associated with low levels of depression, anxiety and substance use. The PPI-2 is a composite score consisting of Machiavellian Egocentricity, Rebellious Nonconformity, Blame Externalization, and Carefree Nonplanfulness, and higher scores reflect a tendency for substance abuse, reckless impulsivity, self-centeredness, and a heightened risk for major Axis 1 disorders including anxiety disorders.

The Generalized Anxiety Disorder 7-item (GAD-7) scale (23), a brief self-report questionnaire, was used to assess generalized symptoms of anxiety, and the Addiction Severity Index-lite (ASI-lite) (24, 25) was used to assess past 30-day substance use and criminal history. The criminal history data used in this study included questions relating to the number of arrests, which were grouped in three categories: Acquisitive Offenses, which included shoplifting, forgery and burglary/larceny; Drug Offenses, which included selling or acquiring drugs; and Violent Offenses, which included assault, rape, homicide, and robbery charges. Data on the total number of convictions and months of incarceration were also collected. Participants were informed that disclosure of certain offenses (e.g., child/elder abuse, homicide) would be reported to authorities.

### MRI Imaging Acquisition

Imaging was performed on a subset of participants (18 controls and 16 MA) on a 3 Tesla Siemens TIM Trio MRI scanner (**Table 1**). A localizer scan was acquired in order to guide slice alignment during anatomical and functional scans. A T_2_*- weighted echo-planar image (EPI) was acquired (24 slices, 4 mm thick, gap width = 1 mm, TR/TE/α = 2,000 ms/40 ms/80°, matrix=128 × 128, FOV=240 mm^2^, 170 volumes, in-plane pixel size of 1.875 mm^2^) while subjects stared at a white cross on a black screen. One high-resolution T1-weighted anatomical magnetically prepared rapid acquisition gradient echo (MPRAGE; 144 slices 1 mm thick, TR/TE/TI/α = 2,300 ms/4.38 ms/1,200 ms/12°, FOV = 208×256 mm) was acquired for co-registration with functional images and statistical overlay.

**Table 1:** Participant characteristics included in imaging analysis.

	Control Group (n = 18)	MA Group (n= 16)	p-value
Age (years) ^a^	37.12 ± 13.92	32.47 ± 9.19	0.281
Sex (M/F) ^b^	11/7	11/5	0.287
Education	14.29 ± 1.69	12.33 ± 1.18	0.001
Number of smokers ^b^	6	14	0.001
PPI- Total	46.88 ± 8.18	54.33 ± 7.17	0.011
PPI-1	154.06 ± 25.02	160.40 ± 21.14	0.448
PPI-2	186.53 ± 18.35	209.73 ± 32.89	0.018
Machiavellian Egocentricity	44.71 ± 7.47	50.00 ± 13.35	0.170
Rebellious Non-conformity	48.82 ± 6.77	52.67 ± 7.20	0.130
Blame Externalization	46.59 ± 8.18	52.67 ± 7.92	0.042
Carefree Non-planning	46.41 ± 9.17	54.50 ± 14.36	0.067
Social Influence	50.53 ± 9.20	52.00 ± 10.80	0.680
Fearlessness	49.00 ± 11.76	57.47 ± 8.81	0.030
Stress Immunity	54.53 ± 9.81	50.93 ± 9.67	0.306
Cold Heartedness	47.18 ± 9.93	48.13 ± 9.09	0.779
Total Convictions	0.53 ± 0.80	14.40 ± 15.57	0.001
Acquisitive offenses	0.12 ± 0.33	8.27 ± 12.47	0.011
Drug offenses	0.18 ± 0.39	3.87 ± 6.64	0.029
Violent offenses	0.24 ± 0.44	1.13 ± 3.04	0.238
Months Incarcerated	2.06 ± 7.27	35.87 ± 49.85	0.010
Anxiety Score (GAD-7)	2.41 ± 2.45	4.73 ± 4.45	0.073
Age of MA first use		18.73 ± 4.38	
Years of MA use		9.133 ± 5.76	
Average use (grams)/day		1.59 ± 1.09	

a Data shown are means ± Standard Deviations.

b Data analyzed with Chi-squared test (X2).

### Resting-State Processing and Group-Level Analyses

Image analysis was performed using FSL 5.0.2.1 (www.fmrib.ox.ac.uk/fsl). Images were skull-stripped, spatially smoothed (5 mm FWHM Gaussian kernel) and realigned to compensate for motion (26). Automatic Removal of Motion Artifacts (AROMA) was then used to reduce motion-induced signal variation using independent component analysis (ICA) with a classifier that uses two temporal and two spatial features to remove motion artifacts. Images were further pre-processed to include additional nuisance regressors: average signal of cerebrospinal fluid and white-matter, and two metrics of motion-related artifact, specifically motion scrubbing with frame-wise displacement and a combination of the temporal derivative of the time series and root-mean-squared variance over all voxels (27) and high-pass temporal filtering (100s). Global signal regression was not applied. The EPI images were registered to the high-resolution MPRAGE image and then into standard Montreal Neurological Institute space, using a 12-parameter affine transformation. An anatomically-defined region of interest (ROI) from the Harvard-Oxford Subcortical atlas of the ventral striatum was used as a seed. The seeds were transformed into each subject's native space by applying the inverted transformation matrix of EPI to MPRAGE to standard space. The mean time series across all voxels within the striatum seed from pre-processed images were used as covariates in separate whole-brain, voxel-wise resting-state correlation analyses. For between-group mixed-effects analyses, PPI-R Total scores were included as regressors to test PPI-R by group interactions in whole-brain voxel-wise RSFC analyses with the ventral striatal seed. All whole-brain functional MRI (fMRI) statistics were corrected for multiple comparisons by using cluster-correction with voxel height threshold of p < 0.001 and cluster significance of p < 0.05.

### Statistical Analysis

Student's t-tests, chi-squared tests, and Fisher's exact tests, where appropriate, were used to compare groups in baseline demographics, clinical variables and criminal history (**Table 2**). Parameter estimates from voxels within significant clusters of activation resulting from group and PPI-R Total score interactions were extracted to examine group interactions with RSFC and PPI-R latent factors of PPI-1 and PPI-2 in SPSS 22. Hierarchical multiple regression analysis was conducted within the MA group to examine the influence of PPI-2, PPI-1, years of MA use, average amount of daily MA use and anxiety on criminal offenses, with a second model incorporating RSFC connectivity values.

**Table 2:** Participant characteristics.

	Control Group (n = 38)	MA Group (n= 33)	p-value
Age (years) ^a^	34.61 ± 11.52	32.39 ± 7.98	0.358
Sex (M/F) ^b^	26/12	25/8	0.493
Education	14.16 ± 2.06	11.94 ± 1.56	0.000
Number of smokers ^b^	15	31	0.001
PPI- Total	48.14 ± 8.432	55.82 ± 9.10	0.001
PPI-1	157.94 ± 20.67	157.55 ± 19.72	0.935
PPI-2	186.44 ± 26.55	217.09 ± 34.48	0.000
Machiavellian Egocentricity	45.61 ± 9.61	51.21 ± 12.55	0.040
Rebellious Non-conformity	49.14 ± 8.82	54.58 ± 9.85	0.018
Blame Externalization	47.94 ± 10.27	56.52 ± 9.93	0.001
Carefree Non-planning	43.75 ± 9.98	54.79 ± 11.94	0.000
Social Influence	52.03 ± 8.48	52.67 ± 10.64	0.783
Fearlessness	50.83 ± 9.88	56.45 ± 9.39	0.018
Stress Immunity	55.08 ± 8.98	48.42 ± 9.79	0.004
Cold Heartedness	47.78 ± 9.84	48.91 ± 9.27	0.625
Total Convictions	0.55 ± 0.76	11.64 ± 14.79	0.000
Acquisitive offenses	0.13 ± 0.41	7.15 ± 13.21	0.002
Drug offenses	0.18 ± 0.46	3.00 ± 4.77	0.001
Violent offenses	0.16 ± 0.37	0.79 ± 2.10	0.074
Months Incarcerated	2.38 ± 7.37	44.45 ± 65.38	0.000
Anxiety Score (GAD-7)	2.13 ± 2.65	5.39 ± 4.75	0.001
Age of MA first use		18.58 ± 6.04	
Years of MA use		10.30 ± 6.58	
Average use (grams)/day		1.57 ± 1.16	

a Data shown are means ± Standard Deviations.

b Data analyzed with Chi-squared test (X2).

## Results

The healthy control group includes 38 subjects (12 women/26 men, 15 smokers, mean ± SD age of 34.61 ± 11.52 years). The MA group includes 33 subjects (8 women/25 men, 31 smokers, mean ± SD age of 32.39 ± 7.98 years), who report using MA for a mean ± SD of 10.30 ± 6.58 years and 1.57 ± 1.16 grams per day and abstinent for 52.18 ± 22.18 days. There are no significant group differences in age or sex but there are significant differences in education (p < 0.001), cigarette smoking status (p = 0.001), and levels of anxiety (p = 0.001) (**Table 2**). There are significant group differences in PPI-R Total scores (p < 0.001) and PPI-2 scores (p < 0.001), with the MA group showing higher scores, and no group differences in PPI-1 scores (p = 0.935). Post-hoc tests of group differences on PPI-R subscales show group differences in 6 of the 8 PPI-R subscales (p < 0.05) (**Table 2**). The MA group report more total convictions and months of incarceration than the control group (p's < 0.001) and report more acquisitive and drug offenses (p's < 0.002) (**Table 2**). Exploratory analysis of sex differences show no significant differences within the MA group for years or amount of daily MA use. There were also no significant effect of sex or interactive effects of sex and group on PPI-R Total, PPIR-1 or PPIR-2 scores.

### RSFC and PPI-R Total Scores

**Figure 1:**
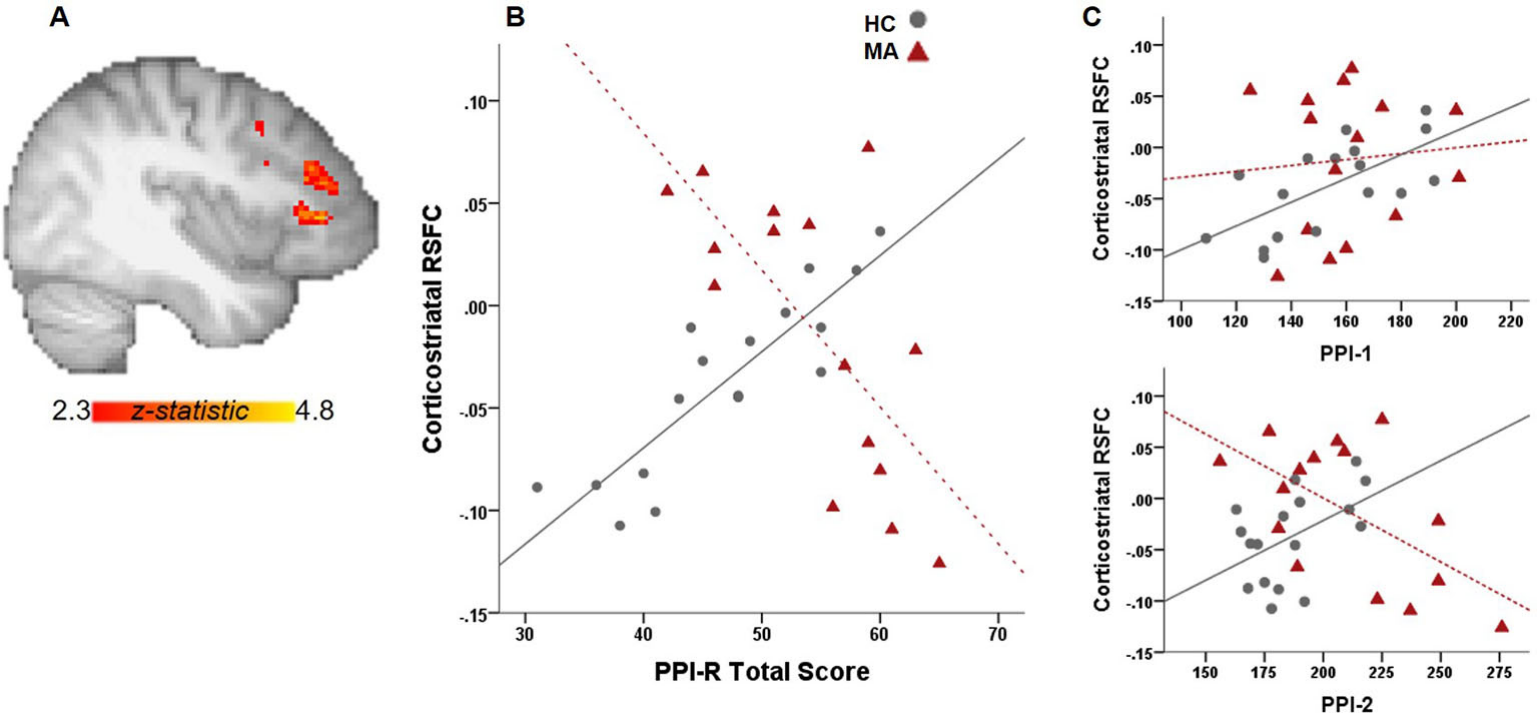
Relationship between PPI total scores and RSFC. **(A)** Whole-brain connectivity analysis with a ventral striatal seed show significant group interactions with PPI-R total scores on corticostriatal connectivity. **(B)** Scatter plot illustrates the relationship between RSFC and PPI-R total scores for each group. **(C)** Significant group interactions on corticostriatal RSFC with PPI-R subscales.

Independent of PPIR scores, the MA group exhibit greater RSFC between the ventral striatum and right middle frontal gyrus than the control group (whole-brain corrected). For group analyses with PPIR- Total scores, there is a significant Group x PPI-R Total score interaction where the MA group exhibits a negative relationship between PPI-R Total scores and RSFC between ventral striatum and middle and inferior frontal gyrus, left anterior insula and cingulate gyrus with the control group showing the opposite effect (whole-brain corrected, **Figure 1**). The connectivity values from the functional ROI of the middle and inferior frontal gyrus show significant group interactions (p = 0.002) with PPI-2 (**Figure 1**), with a negative relationship in the MA group and a positive relationship in the control group. Within the MA group, the negative relationship remains significant after controlling for days abstinent.

### PPI-R Scores, Criminal Convictions, and RSFC

**Figure 2:**
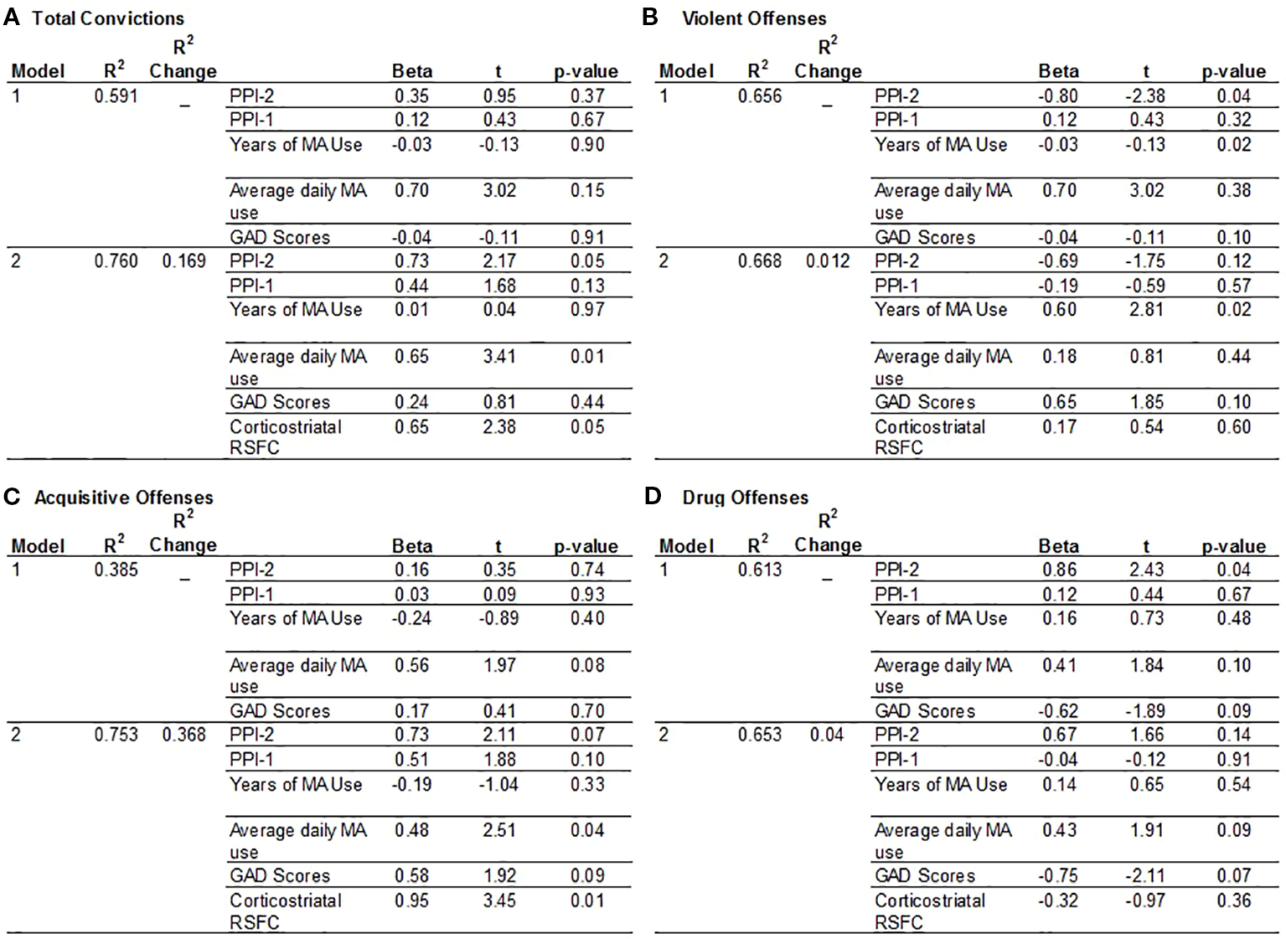
Hierarchical multiple regression analysis to predict criminal offenses. The model includes factors hypothesized to contribute to a history of criminal convictions in the MA group. Model 1 includes PPI-1 and PPI-2 scores, Years of MA use, Average daily MA use and GAD-7 anxiety scores. Model 2 includes variables listed in Model 1 plus corticostriatal RSFC values. **(A)** Total Convictions: RSFC values improved the model by 1.69%. The most significant predictors are PPI-2 scores, daily MA use and corticostriatal RSFC. **(B)** Violent Offenses: RSFC values only improved the model by 1.2% and the most significant predictors of violent offenses are PPI-2 and years of MA use. **(C)** Acquisitive Crimes: RSFC values increased the model by 36.8.5% and indicates that greater daily MA use and greater corticostriatal RSFC are significant predictors of acquisitive crimes. **(D)** Drug Offenses: The independent variables accounted for the least amount of variance associated with drug offenses with greater PPI-2 scores associated with more drug offenses.

There is a significant group interaction with PPI-R Total scores on total number of convictions (p = 0.034), however the results did not retain significance after excluding a MA participant with a high number of convictions and PPI total score. The limited range in the control group for total number of convictions, and for acquisitive, drug and violent offenses, however, limits the interpretation of any group interactions on criminal offenses. The hierarchical multivariate analysis of criminal history is, therefore, only examined in the MA group after excluding an outlier. For total convictions, ventral striatal-PFC RSFC is significantly associated with total number of convictions and the addition of RSFC in the model increases R^2^ by 0.169. PPI-2 and amount of average daily MA use are both significant predictors for total number of convictions after accounting for ventral striatum-PFC RSFC, where an increase in PPI-2 scores and daily MA use are associated with a greater number of convictions (**Figure 2A**). Ventral striatum-PFC RSFC does not account for additional variability in violent offenses and only accounts for a.012 change in R^2^ value. However, PPI-2 and years of MA use contribute to the number of violent offenses, where lower PPI-2 scores and more years of MA use is associated with more violent offenses but only years of MA use remains significant after accounting for RSFC (**Figure 2B**). For acquisitive crimes, ventral striatum-PFC RSFC improves model fit and accounts for 37% more variability than the model with only PPI-1, PPI-2, years, and amount of MA use and anxiety scores. An increase in RSFC and an increase in the amount of daily MA use are associated with higher numbers of acquisitive offenses (**Figure 2C**). For drug offenses, PPI-2 is only a significant predictor when RSFC values are excluded from the model (**Figure 2D**).

## Discussion

This study examined the extent to which psychopathic personality traits, MA use and associated brain connectivity confers predisposition to criminal involvement. We used the PPI-R, a self-report measure of psychopathy, and found that the MA group exhibited higher levels of psychopathy and reported significantly greater numbers of criminal offenses and convictions. The impulsive antisocial dimension of psychopathy (PPI-2) was a significant predictor in total convictions, violent offenses, and drug offenses, while amount of daily MA use were factors in total convictions and acquisitive crimes and years of MA use were associated with violent offenses. We also report different patterns of corticostriatal connectivity as a function of psychopathy total scores between MA-dependent individuals and controls with similar patterns for PPI-2, the impulsive antisocial dimension of psychopathy. Corticostriatal RSFC, however, was only a significant predictor for total convictions and acquisitive crimes but did increase the predictive validity of PPI-2 for total convictions.

Similar to other studies showing greater RSFC in MA groups compared to control groups (5, 17, 18), the MA group exhibit greater RSFC between ventral striatum and right middle frontal gyrus compared to controls independent of PPI-R scores. These results are consistent with studies that suggest MA-induced abnormalities in corticostriatal circuits, which may manifest in poor decision-making, impulsivity, and behavioral phenotypes associated with addiction. The results with PPI-R total scores are also consistent with other studies that report heightened levels of psychopathy in drug-dependent groups (1, 28), and here we extend the literature to show that MA use disorder is also associated with significantly higher levels of psychopathy total scores and in the impulsive antisocial dimension of psychopathy (PPI-2). The MA group also exhibited significantly more criminal offenses than controls, which is in line with a report that MA use is a significant predictor in criminal behavior and recidivism (7). There were no significant interactions of group and psychopathy on total number of convictions; however, the limited range of convictions in the control group prevents definitive interpretation.

There is a high prevalence of MA addiction among those incarcerated (Office of National drug control policy 2006), however, the relationship between MA use, psychopathic traits and crime is complex and moderated by a number of factors. Here we investigated the relationship between psychopathy and functional connectivity of the ventral striatum. Higher total psychopathy scores were significantly related to weaker corticostriatal connectivity in the MA group, while the opposite was true for the control group. In addition, the impulsive antisocial dimension of psychopathy (PPI-2) showed similar group interactions. As cortical modulation of striatal activity is thought to promote adaptive and goal-directed behavior, abnormalities in corticostriatal connectivity associated with psychopathic traits in MA-use disorder may promote impulsivity and enhance maladaptive behavior in those with higher levels of psychopathy. This is consistent with other studies showing that corticostriatal and striatal connectivity is related to cognitive impulsivity and reward-driven behavior in MA use disorder (5, 18) and to temporal discounting in psychopathy (9).

This study extends previous studies in MA use disorder by showing that PPI-R scores and corticostriatal RSFC are significant predictors for the number of total convictions and acquisitive crimes. This agrees with a study showing that psychopathy is related to weaker corticostriatal RSFC, which is a predictor for a greater number of criminal convictions (9). In addition, we extend these results to show that average daily MA use contributes to total convictions. Similarly, average daily MA use and corticostriatal RSFC predict in an increase in acquisitive crimes. Interestingly, the regression shows that an increase in average MA use and stronger corticostriatal RSFC predicts a greater number of acquisitive crimes. As offenders of acquisitive crimes exhibit better cognitive function indexed by performance on measures of working memory, mental flexibility, capacity to plan action, and control of interference (29), greater corticostriatal RSFC coupled with an increase in MA use may contribute to impulsive behavior and enable those with MA-use disorder to execute goal-directed action. Although speculative, this notion is supported by results showing that MA users with greater striatal RSFC exhibit greater cognitive impulsivity (18), which differs from behavioral impulsivity in that it requires mental control, the ability to shift mental set or reasoning and is required in acquisitive crimes to a greater extent than violent behavior (29). In addition, there is an interaction between cognitive impulsivity and executive function, where higher cognitive impulsivity and low cognitive functioning is associated with more violent behaviors, while acquisitive crimes are linked to high cognitive impulsivity and functioning (29, 30). These results are interesting in the context of discussions on strengthening corticostriatal connections to promote adaptive decision-making in addiction. Although the goal of various treatment approaches is to enhance executive function through talk therapy or through functional connections with pharmacotherapies, these results highlight the importance of addressing impulsive and antisocial personality traits and the amount of MA use that could reinforce maladaptive behavior.

Consistent with studies showing that psychopathy predict higher levels recidivism (31), our results show that the impulsive antisocial dimension of psychopathy are associated with drug and violent offenses. Interestingly, the significance of PPI-2 as a predictor of these offenses are no longer significant after accounting for corticostriatal connectivity. However, years of MA use remains significantly associated with violent offenses after controlling for RSFC. Violent aggressive crimes have been linked to the pharmacological effects of MA, as psychosis and paranoia associated with intoxication leads to behavioral inhibition and criminal activity (11). Although this study did not assess whether violent offenses were associated with intoxication, we show that years of MA use is only associated with violent offenses, which accords with the association between violent offenses and more severe histories of substance use (32). Despite a number or studies reporting the link between anxiety and criminal activity and the link between MA use and anxiety, this study found no significant effects of anxiety on criminal offenses or convictions. Although dimensions of psychopathy are associated with anxiety (21), there is limited work describing the in interaction of anxiety and crime in MA use disorder and future studies addressing these two personality factors may provide important insight to reduce criminal offenses and recidivism and improve treatment outcomes in MA use disorder.

Criminal behavior in MA dependence is, however, conditional on many factors (6), some of which may be premorbid to MA use. The use of MA and related neural deficits can also contribute to criminal behavior through the lack of inhibitory/behavioral control, maladaptive decision-making processes and difficulties in interpersonal communication and emotion regulation (4, 6). However, it is unclear the extent to which MA use, independent of PPI-R is a factor in criminal offenses. A post-hoc analyses was therefore conducted to examine the regressions without PPIR scores, which show that daily MA use is still associated with Total convictions and Acquisitive offenses, while years of MA use is still significantly associated with Violent offenses. However, a number of personal and contextual characteristics accompany addiction and motivate criminal behavior, gaining an understanding of neurobiological factors paired with personality and environmental variables may aid in developing future effective psycho- and pharmacotherapies.

## Limitations

Although this study begins to detail the complex interactions of MA use, neurobiological dimensions of psychopathy to predict criminal behavior, it is not without limitations. Self-reports were used to assess criminal offenses and it is possible that the number of offenses were either inflated or underreported. Future studies should consider obtaining official records for accurate criminal history data. In addition, a more detailed description of violent offenses would help dissociate violent behavior resulting from MA intoxication from predisposing factors for violence. The small sample sizes preclude the examination of sex and other demographic variables in the analysis and results should be interpreted with caution. The limited number of offenses in the control group also limits the interpretation of group interactions on criminal history and future studies would benefit from recruiting control participants with greater variability in criminal convictions. In addition, differentiating first-time offenses from that of re-offenses may provide insight aimed to reduce recidivism in MA use disorder. Lastly, it cannot be determined to what extent brain connectivity, psychopathy and anxiety is premorbid to MA use or to what extent MA use exacerbates these outcome variables.

## Conclusions

Despite these limitations, these results extend previous studies examining the association between brain function and psychopathy by providing evidence for the interaction of substance use and psychopathic traits on brain connectivity. This study also highlights the complex relationship between MA use, personality traits and criminal behavior and suggests that although brain function is an important component in a subset of criminal activity, therapies should focus on impulsivity/antisocial dimensions of personality to reduce criminal convictions.

## Data Availability Statement

The datasets generated for this study are available on request to the corresponding author.

## Ethics Statement

The studies involving human participants were reviewed and approved by VAPORHCS and Oregon Health & Science University Institutional Review Boards. The patients/participants provided their written informed consent to participate in this study.

## Author Contributions

WH designed and implemented the study. LD and HM managed and oversaw the study implementation. MK conducted the analysis and drafted the manuscript. MK, MJ, and AH contributed to data interpretation. SS participated in data collection. All authors took part in the revision of the manuscript and approved the article.

## Funding

This work was supported in part by the Department of Veterans Affairs Clinical Sciences Research and Development Merit Review Program, I01 CX001558-01A1 (WFH); Department of Justice 2010-DD-BX-0517 (WFH); National Institute on Drug Abuse P50DA018165 (WFH); Oregon Clinical and Translational Research Institute, 1 UL1 RR024140 01 from the National Center for Research Resources, a component of the National Institutes of Health and National Institute of Health Roadmap for Medical Research. Dr. Kohno was supported by National Institute on Drug Abuse T32 DA007262, National Institute on Alcohol Abuse and Alcoholism T32 AA007468, Department of Veterans Affairs Clinical Sciences Research and Development Career Development Award IK2CX001790, Oregon Health & Science University Collins Medical Trust Award APSYC0249, Medical Research Foundation of Oregon APSYC0250, and the Center for Women’s Health - Circle of Giving APSYC0287.

## Disclaimer

The contents of this paper do not represent the views of the U.S. Department of Veterans Affairs or the United States Government.

## Conflict of Interest

The authors declare that the research was conducted in the absence of any commercial or financial relationships that could be construed as a potential conflict of interest.
